# The use of warfarin in veterans with atrial fibrillation

**DOI:** 10.1186/1471-2261-4-18

**Published:** 2004-10-21

**Authors:** Dawn M Bravata, Karen Rosenbeck, Sue Kancir, Lawrence M Brass

**Affiliations:** 1 Clinical Epidemiology Research Center, VA Connecticut Healthcare System, 950 Campbell Avenue 11C-2, West Haven, CT 06516, USA; 2 Department of Internal Medicine, VA Connecticut Healthcare System, 950 Campbell Avenue, West Haven, CT 06516, USA; 3 Department of Quality Management, VA Connecticut Healthcare System, 950 Campbell Avenue, West Haven, CT 06516, USA; 4 Department of Neurology, VA Connecticut Healthcare System, 950 Campbell Avenue, West Haven, CT 06516, USA; 5 Yale University School of Medicine, 333 Cedar Street, Room IE-61 SHM, New Haven, CT 06520-8088, USA

**Keywords:** Atrial fibrillation, warfarin, anticoagulation, preventive medicine, guideline adherence

## Abstract

**Background:**

Warfarin therapy is effective for the prevention of stroke in patients with atrial fibrillation. However, warfarin therapy is underutilized even among ideal anticoagulation candidates. The purpose of this study was to examine the use of warfarin in both inpatients and outpatients with atrial fibrillation within a Veterans Affairs (VA) hospital system.

**Methods:**

This retrospective medical record review included outpatients and inpatients with atrial fibrillation. The outpatient cohort included all patients seen in the outpatient clinics of the VA Connecticut Healthcare System during June 2000 with a diagnosis of atrial fibrillation. The inpatient cohort included all patients discharged from the VA Connecticut Healthcare System West Haven Medical Center with a diagnosis of atrial fibrillation during October 1999 – March 2000. The outcome measure was the rate of warfarin prescription in patients with atrial fibrillation.

**Results:**

A total of 538 outpatients had a diagnosis of atrial fibrillation and 73 of these had a documented contraindication to anticoagulation. Among the 465 eligible outpatients, 455 (98%) were prescribed warfarin. For the inpatients, a total of 212 individual patients were discharged with a diagnosis of atrial fibrillation and 97 were not eligible for warfarin therapy. Among the 115 eligible inpatients, 106 (92%) were discharged on warfarin.

**Conclusions:**

Ideal anticoagulation candidates with atrial fibrillation are being prescribed warfarin at very high rates within one VA system, in both the inpatient and outpatient settings; we found warfarin use within our VA was much higher than that observed for Medicare beneficiaries in our state.

## Background

Warfarin therapy is highly effective for the prevention of ischaemic stroke in atrial fibrillation [1]. Despite the accepted benefit of warfarin therapy, several reports have indicated that warfarin therapy is underutilized even in ideal anticoagulation candidates with atrial fibrillation. Most studies have reported rates of use between 13–60% [2-8]. For example, a national study of inpatient Medicare beneficiaries with atrial fibrillation demonstrated that approximately 55% of patients were discharged on warfarin [9].

Many of the previous studies about the use of warfarin in atrial fibrillation have focused on the prescription of warfarin on discharge from an acute hospitalization. Since some patients may be discharged from the hospital with a plan to begin warfarin therapy as an outpatient, these prior studies may have underestimated the use of warfarin for patients with atrial fibrillation. The Veterans Affairs (VA) Healthcare System is a useful setting for studying the use of warfarin therapy in both the inpatient and outpatient arenas because the electronic medical record contains prescription medication data as well as the inpatient and outpatient medical records (including progress notes, laboratory data, radiology reports, and other consult reports).

The objective of this study was to examine the use of warfarin in both inpatients and outpatients with atrial fibrillation within a VA setting. Specifically, we used the same methodology as the Medicare Health Care Quality Improvement Program's National Stroke Project – Atrial Fibrillation [10], so that we could compare rates of warfarin use in ideal anticoagulation candidates with atrial fibrillation from one VA system to those in the private sector.

## Methods

We assembled two retrospective cohorts of patients to evaluate both inpatients and outpatients with atrial fibrillation. The medical records of both the inpatients and the outpatients were reviewed to confirm the diagnosis of atrial fibrillation, to identify any exclusion criteria, and to determine if patients were being prescribed warfarin.

### Diagnosis of atrial fibrillation

Using the criteria developed by the Medicare Health Care Quality Improvement Program's National Stroke Project – Atrial Fibrillation [10], a physician's documentation of the diagnosis of atrial fibrillation was required for inclusion (electrocardiogram data were not used to make the diagnosis of atrial fibrillation). For the outpatients, Physicians' Current Procedural Terminology (CPT) codes were used to identify potential patients with a diagnosis of atrial fibrillation. For the inpatients, the International Classification of Diseases, 9th Revision, Clinical Modification (ICD-9-CM) discharge diagnosis code (427.31) was used to identify potential patients with a diagnosis of atrial fibrillation. Chart review was used to confirm the diagnosis for both the inpatients and the outpatients; a physician's documentation of atrial fibrillation in a progress note, a consult note, the discharge summary, or the problem list was needed for confirmation.

Medical record abstraction was conducted by two of the authors (KR, SK) using standard definitions. All of the exclusions were reviewed by three of the authors (KR, SK, DMB), a sample of the charts of patients with an exclusion criteria was re-abstracted (DMB), and any disagreements were resolved by consensus.

### Cohort descriptions

The outpatient cohort included all of the patients seen in the outpatient clinics of the VA Connecticut Health Care System during the month of June 2000 with a diagnosis of atrial fibrillation. Outpatient clinics include both primary care and subspecialty clinics. Some of these clinics have a particular interest in the care of patients with atrial fibrillation such as cardiology and anti-coagulation clinics, however, most of the clinics do not have such a special interest (e.g., mental health, physical therapy, dermatology, endocrinology).

The inpatient cohort included all patients discharged from the VA Connecticut Health Care System West Haven Campus with any discharge diagnosis of atrial fibrillation (primary or secondary diagnosis) during the period of October 1, 1999 through March 31, 2000. Some of the patients in the inpatient cohort were readmitted during our study period; this report includes data from individual patients for their first hospital stay.

### Exclusion criteria

The Medicare Health Care Quality Improvement Program's National Stroke Project – Atrial Fibrillation project developed a set of exclusion criteria to identify ideal candidates for warfarin therapy; we used this exclusion criteria for the current study. Patients were excluded if they met one or more of the following: current sinus rhythm; bleeding disorder; endocarditis or pericarditis (within 6 months); seizures; intracranial hemorrhage; intracranial surgery or biopsy; lone atrial fibrillation; dual chamber pacemaker; alcohol or drug abuse; allergy to warfarin; hepatic failure; schizophrenia or active psychotic disorder; comfort care or terminal illness with life expectancy less than 6 months; un-repaired intracranial aneurysm; extensive metastatic cancer; brain cancer; malignant hypertension; peptic ulcer disease; hemorrhage; documentation that the patient refused warfarin therapy; prior complication or allergy related to past use of warfarin; or physician documentation of a rationale for not prescribing warfarin, including risk for bleeding, risk for falls, mental status impairment, liver disease, arthritis requiring non-steroidal anti-inflammatory medications or aspirin, pending surgery or other invasive procedure, terminal illness, patient's inability to obtain necessary blood work, or history of patient's non-adherence to warfarin [10]. Patients in intermittent or paroxysmal atrial fibrillation were included in the study, however, patients who were noted to be in current sinus rhythm and for whom atrial fibrillation was not a current problem were not included. For example, a patient with atrial fibrillation in the setting of an acute myocardial infarction or post-coronary bypass grafting for whom the atrial fibrillation was not a current medical problem was not included.

### Warfarin prescription

For all of the patients, warfarin prescription was determined from the medical record. Patients receiving warfarin from the VA pharmacy were readily identified from the VA pharmacy component of the medical record. Patients receiving warfarin privately were identified from the progress notes. For inpatients, warfarin prescription was evaluated at the time of discharge from the hospital. For outpatients, warfarin prescription was evaluated at the time of examination of the medical record. Each outpatient medical record was examined in detail to determine the presence or absence of warfarin prescription. For those outpatients patients in whom this determination was difficult or if the data collector had a question about the patient, then the medical record was examined again by three of the authors to determine the presence or absence of warfarin prescription. This study received Institutional Review Board approval.

### Statistical analysis

Student's t-tests were used to compare dimensional variables, and Fisher Exact and chi-square tests were used to assess binary variables. Two-sided p-values <0.05 were considered to be statistically significant. Exact binomial 95% confidence interval were calculated for the proportions of patients using warfarin in the inpatient and outpatient cohorts. The SAS System software release 6.12 (Cary, N.C.) was used for data analysis.

## Results

### Outpatient cohort

A total of 538 patients from the VA Connecticut outpatient clinics were identified as having a diagnosis of atrial fibrillation (age: 74.0 years mean ± 8.3 standard deviation; 529 [98%] men). Of these, 73 patients had one or more contraindication to anticoagulation (Table 1). Of the 465 eligible patients, 455 (98%; 95%CI 96–99%) were prescribed warfarin.

**Table 1:** Exclusion Criteria*

**Characteristic**	**Inpatients**	**Outpatients**
	N = 212	N = 538
	N (%)†	N (%)†
Sinus rhythm	32 (15)	42 (8)
Death	21 (10)	1 (0.2)
History of gastrointestinal hemorrhage	18 (8)	6 (1)
Fall risk	14 (7)	4 (0.7)
Pacemaker	4 (2)	9 (2)
Lone atrial fibrillation	4 (2)	1 (0.2)
Terminal illness	4 (2)	0 (0)
Patient refused	3 (1)	6 (1)
History of intracranial hemorrhage	3 (1)	3 (0.6)
Transfer to outside facility	3 (1)	0 (0)
Multi-infarct dementia in comfort care patients	2 (0.9)	0 (0)
Warfarin held for procedure or surgery	2 (0.9)	0 (0)
Warfarin allergy	1 (0.5)	0 (0)
Failed to comply with warfarin protocol, warfarin stopped	1 (0.5)	1 (0.2)
History of seizures	1 (0.5)	0 (0)
Elective admission to begin sotalol and discontinue warfarin	1 (0.5)	0 (0)
Previous bleeding on warfarin	0 (0)	1 (0.2)

*These exclusion criteria were taken directly from the Medicare Health Care Quality Improvement Program's National Stroke Project – Atrial Fibrillation.^10^

^†^Note: some patients had more than one reason for not being prescribed warfarin.

The ten patients who were not prescribed warfarin did not differ from those who received warfarin with respect to age (mean age ± standard deviation; no warfarin: 76.6 ± 11.5, warfarin: 73.9 ± 8.1; p = 0.3). Among the ten patients who were not prescribed warfarin: one was a dialysis patient who received his medical care primarily from private physicians outside of the VA, he was eventually placed on warfarin; the medical record of one 90-years-old patient, who also received the majority of his health care from private physicians, indicated that his private physician had elected not to prescribe anticoagulation "because of age"; one patient had a history of alcohol use; and in the remaining 7 patients there was no documentation of a reason for why the warfarin had not been prescribed (3 of the 7 patients were receiving the majority of their medical care outside of the VA).

### Inpatient cohort

A total of 212 individual patients were discharged with a diagnosis of atrial fibrillation (age: 72.9 years mean ± 9.9 standard deviation; 211 [99.5%] males). During the admission these 212 patients, 17 died during their hospitalization, 3 were transferred to a facility outside of the VA Connecticut Healthcare System, and 77 had one or more contraindication to anticoagulation. Of the 115 remaining eligible patients, 106 (92%; 95%CI 86–96%) were discharged on warfarin.

The nine patients who were not prescribed warfarin did not differ from those who were prescribed warfarin with respect to age (mean age ± standard deviation; no warfarin: 76.4 ± 7.4, warfarin: 72.6 ± 7.9; p = 0.2). To determine if some of the eligible inpatients who were not discharged on warfarin later received warfarin in the outpatient setting, we examined the outpatient records of the nine inpatients who were not discharged on warfarin: 5 died; 3 no longer receive care at our medical center (and no medication data were available); and for 1 patient, the medical record stated that he was offered warfarin therapy but that he refused to accept it. Similarly, we evaluated a sample of 50 eligible patients who had been discharged on warfarin therapy and examined their warfarin use post-discharge: 7 no longer receive care at our medical center (and no medication data are available); 4 were taken off of warfarin (3 because they were cardioverted as outpatients, and for 1 patient the warfarin was discontinued after an episode of bright red blood per rectum); and 2 patients have died.

Among the eligible inpatients 12/115 (10%) had history of prior stroke or transient ischemic attacks; 3/12 (25%) were not prescribed warfarin on discharge. No reasons were documented for why these patients were not given warfarin.

### Unique patients

There was overlap between the inpatient and outpatient cohorts such that a total of 722 unique patients were identified among the 212 inpatients and the 538 outpatients.

## Discussion

We found high rates of warfarin prescription in ideal anticoagulation candidates with atrial fibrillation treated within this VA system. A total of 561 of 580 ideal anticoagulation candidates (97%) were prescribed warfarin: 98% of ideal outpatient anticoagulation candidates and 92% of ideal inpatient anticoagulation candidates. These rates of warfarin use for atrial fibrillation are substantially higher than those reported previously from private sector academic and community hospitals. For example, as part of the Medicare Health Care Quality Improvement Program's National Stroke Project – Atrial Fibrillation, medical records were reviewed from a random sample of Medicare beneficiaries, hospitalized during the period 1998–1999, with any discharge diagnosis of atrial fibrillation from each state [9,10]. The exclusion criteria for the Medicare medical record review were the same as those used for the current study and were developed to select a cohort of atrial fibrillation patients who were "ideal" candidates for oral anticoagulation because they do not have any contraindications to oral anticoagulation [10]. Therefore, one would expect that the rates of warfarin use would be higher in ideal anticoagulation candidates than in a general population of patients with atrial fibrillation. Overall, the rate of warfarin prescription for ideal anticoagulation candidates with atrial fibrillation patients by state ranged from 31–65%, with a median of 55% in the Medicare study [9]. In Connecticut, 57% of eligible atrial fibrillation inpatients were discharged on warfarin [9]. Therefore, the inpatient rates observed in the current study of 90–92% are much higher than those observed for Medicare beneficiaries using similar methodology.

We report the anticoagulation rates from one VA healthcare system, and our findings may not be generalizable to other VA healthcare systems or to non-VA hospitals. Specifically, our results may not be generalizable to women with atrial fibrillation. Furthermore, given that the out-patient cohort for this study was obtained from a one-month sample, we may have selected patients who are more likely to be seen at an out-patient clinic, and therefore, our findings may also have limited generalizability to atrial fibrillation patients who do not require or who do not have access to regular out-patient clinical care. Although we have found higher rates of warfarin use than most of the previous studies in this area [2-9], our findings are similar to those reported by Gottlieb and Salem-Schatz [11] who found that 78.8% of atrial fibrillation patients in an HMO setting were receiving warfarin. Our findings are also similar to those reported by Bradley et al. from another VA health care system [12]. Bradley et al. demonstrated that 89% of patients without a contraindication to anticoagulation were prescribed warfarin [12].

Several possible factors might account for the high use of warfarin in VA hospitals. First, the actual rates of warfarin prescription may be higher in VA facilities where anticoagulation clinics are well established, the electronic medical record ensures that all services (primary care and consult services) have access to a patient's medical record, the staff has academic affiliations, and the VA culture embraces quality improvement and medical error reduction initiatives. Within the VA Connecticut Health Care System, pharmacist-directed anticoagulation clinics are available at two sites, in West Haven and Newington, Connecticut. Veterans who choose to obtain warfarin from the VA pharmacy are usually followed at one of these two anti-coagulation clinics. Veterans can elect to purchase warfarin from private pharmacies and have their anticoagulation intensity monitored privately (usually by their private internist or private cardiologist). Second, the higher rate may result from data collection differences between studies. Specifically, given the comprehensive VA electronic medical record, VA-based researchers may be able to identify more contraindications for anticoagulation. No quality improvement projects to increase the use of warfarin for patients with atrial fibrillation in the VA Connecticut Healthcare System were initiated during or immediately prior to the study period. A limitation of the current study is that we were unable to determine the specific reasons for why such a high rate of warfarin use was observed.

The retrospective nature of this study permitted us to evaluate clinical practices without altering physicians' behavior. However, this retrospective chart review may have limitations. First, we may not have identified those patients who are prescribed warfarin by private practitioners and obtain their warfarin from non-VA pharmacies. This would result in even higher rates of warfarin prescription than we have reported.

Second, we assembled our cohort using diagnosis codes for atrial fibrillation and did not use electrocardiographic data. Those patients who had atrial fibrillation by electrocardiogram, but who were not identified as having atrial fibrillation by their clinicians, would not have been included in this study. Because such patients are unlikely to receive warfarin our estimates of warfarin use are higher than would have been observed if we had used electrocardiography to identify atrial fibrillation patients. While many studies of the use of warfarin for atrial fibrillation have also assembled cohorts using diagnosis codes and not electrocardiographic data, the VA-based study by Bradley et al. used electrocardiographic criteria and their findings are similar to ours [12].

Third, some may argue that we excluded patients who would benefit from anticoagulation. For example, patients with atrial fibrillation and numerous other risk factors for stroke might benefit from anticoagulation despite the presence of a contraindication to anticoagulation such as a risk for falls. We chose to use the exclusion criteria developed for the Medicare Health Care Quality Improvement Program's National Stroke Project – Atrial Fibrillation [10] so that we could compare the rates of warfarin prescription observed within one VA system to those seen for Medicare beneficiaries.

Fourth, this study includes a total of 722 unique patients. Although some studies of warfarin use in patients with atrial fibrillation have included similar numbers of patients (e.g., N = 635 in the study of Medicare beneficiaries with ischemic stroke and atrial fibrillation by Brass, et al) [13], many studies have included much larger sample sizes (e.g., N = 11,699 in the study of Medicare beneficiaries with new-onset atrial fibrillation) [14]. Often, the studies with the largest sample sizes were secondary analyses of existing administrative datasets [14]. Future studies should be directed at evaluating the use of oral anticoagulation in veterans with atrial fibrillation using national VA data where both large sample sizes and nationally representative sampling are possible.

## Conclusion

We conclude that high rates of adherence to treatment guidelines regarding the use of anticoagulation in patients with atrial fibrillation can be achieved. Our experience, and that of Bradley et al., indicates that high rates of warfarin use can be achieved across at least two VA settings [12].

## Competing interests

The authors declare that they have no competing interests.

All of the authors are employed by the VA Connecticut Healthcare system. Dr. Bravata is supported by an Advanced Research Career Development Award from the Department of Veteran Affairs Health Services Research & Development Service.

## Authors' contributions

All of the authors contributed to this manuscript, participated in the research design and manuscript preparation. Two of the authors (SK, KR) conduct the data collection. Three of the authors (DMB, SK, KR) reviewed the data and conducted the analyses.

## Pre-publication history

The pre-publication history for this paper can be accessed here:


